# Lessons Learned from a Shared Curriculum on Tobacco Cessation Using a Mixed-Methods Approach

**DOI:** 10.3390/pharmacy11040123

**Published:** 2023-08-02

**Authors:** Nervana Elkhadragy, Robin L. Corelli, Noll L. Campbell, Alan J. Zillich, Karen Suchanek Hudmon

**Affiliations:** 1 School of Pharmacy, University of Wyoming, Laramie, WY 82017, USA; 2 College of Pharmacy, Purdue University, West Lafayette, IN 47907, USA; 3 School of Pharmacy, University of California San Francisco, San Francisco, CA 94143, USA

**Keywords:** pharmacy, pharmacy education, shared curricula, Rogers’ Diffusion of Innovations Theory, tobacco cessation

## Abstract

Although the sharing of curricular content between health professional schools can reduce faculty burden, the literature provides little guidance to support these efforts. The objective of this investigation was to synthesize data from two prior studies to delineate recommendations guiding the future development of shared curricula in health professional education. Applying Rogers’ Diffusion of Innovations Theory as a guiding framework, relevant data were extracted from a two-phase mixed-methods study evaluating the long-term impact of the shared Rx for Change: Clinician-Assisted Tobacco Cessation program. Phase 1, a qualitative study, involved telephone interviews with faculty participants of train-the-trainer workshops conducted between 2003 and 2005. These results informed the development of a phase 2 national survey, administered electronically as a long-term follow-up (13 to 15 years later) with train-the-trainer workshop participants. Results from the two studies were synthesized and summarized, producing seven key recommendations to guide development of shared curricula: (1) appeal to attendees, (2) relate content to clinical practice, (3) deliver live, in-person training, (4) develop high-quality materials, delivered by experts, (5) provide support, (6) meet accreditation standards, and (7) demonstrate effectiveness. Future program developers should consider these recommendations to enhance dissemination, adoption, and long-term sustainability of shared curricular content.

## 1. Introduction

Health professional educators are responsible for improving students’ foundational knowledge and clinical skills. To achieve this goal, high-quality, engaging, evidence-based teaching materials are needed for relevant topics across multiple years of a degree program. Because individual faculty members typically create their own lecture materials, this translates into hundreds of faculty members creating comparable materials on similar topics—an approach that is costly and time-consuming.

One solution to promote quality while reducing faculty burden is the concept of “shared curricula.” There is no universally accepted definition of a “shared curriculum,” thus we define it as an open access, comprehensive curriculum covering a specific topic that is created by a group of content experts, and which evolves over time based on input from faculty utilizing the materials. This paper describes the sharing component, including how a curriculum is shared among faculty members, and the necessity of ongoing updates and support [1,2]. Currently, few shared curricula in pharmacy education (tobacco cessation [3], pharmacogenomics [4], cultural competence [5], and infectious diseases [6]) have been disseminated within or across health professional programs. Faculty educators value having access to shared curricular content and perceive it as a resource for teaching materials that prevents faculty members from reinventing the wheel [7]. While developing shared content is an important first step, it is equally (if not more) important to develop effective methods to ensure its broad-scale dissemination, adoption, and sustainability. The existing literature provides little guidance with respect to these key steps.

Of previously developed shared curricula, the *Rx for Change: Clinician-Assisted Tobacco Cessation* curriculum (https://rxforchange.ucsf.edu, accessed on 28 June 2023) [3] has been in existence for the longest period of time. This national curriculum was developed in 1999 in response to a survey of pharmacists that identified a need for enhanced tobacco-cessation training in pharmacy schools [8]. With funding from the National Cancer Institute, a nationwide dissemination effort was launched for which two faculty members from each pharmacy school in the United States were invited to attend one of five 2.5-day train-the-trainer workshops conducted between 2003 and 2005. Significant effort went toward identifying the most appropriate faculty members to participate, with an emphasis on recruiting one person who could capably teach the biological basis of dependence and another who could teach behavioral aspects of quitting and facilitate pharmacy practice skills laboratories. To maximize participation, travel and expenses were covered with grant funds. A total of 191 participants, representing 89 of 91 pharmacy schools in existence at the time (98%), attended a workshop. The workshop was designed to support the implementation of the Rx for Change teaching materials into the doctor of pharmacy curricula at participating schools of pharmacy [9].

Results from this national initiative have shown long-term sustainability, with an estimated 73% of pharmacy schools using the Rx for Change content as part of their core curriculum 15 years later [10]. This sustainability warranted further exploration, and in 2017–2018 we conducted a qualitative interview study with faculty who had participated in a train-the-trainer workshop. The first interviewee stated, *“I’m not sure what that special ingredient is that makes some of these [workshops] work really well and some of them not.”* This comment inspired us to investigate further why the Rx for Change dissemination workshops were successful and what could be learned from this experience. Although this study was focused on a unique tobacco-cessation program, it can be used as a model and offers important takeaway lessons for other topics that would benefit from shared curricula. Thus, the objective of this report was to synthesize data from our prior studies, described below, and delineate specific recommendations that could inform future shared curriculum development efforts. Such recommendations are intended to facilitate the dissemination, adoption, and long-term sustainability of newly created shared curricula.

## 2. Materials and Methods

Development and dissemination of the Rx for Change program, as well as the long-term follow-up mixed-methods study described here, applied Rogers’ Diffusion of Innovations Theory as a guiding framework [11]. This approach encompasses five domains (“characteristics of the innovation”) that influence adoption of a new program: (1) relative advantage—the degree to which an innovation is perceived by users as better than previous ideas; (2) compatibility—the degree to which an innovation is perceived as being consistent with the values, past experiences, and needs of potential adopters; (3) complexity/simplicity—the degree to which an innovation is perceived as easy to understand and use; (4) trialability—the degree to which experimentation is possible with an innovation; and (5) observability—the ability to see the results of an innovation. By elucidating how participants view the aforementioned five factors, one can attempt to identify key characteristics of an innovation that are associated with its adoption and sustainability [11].

Relevant data were extracted and synthesized from a two-phase mixed-methods study (summarized briefly below; greater detail is provided elsewhere [7,12]) evaluating the long-term impact of the Rx for Change program and its train-the-trainer workshops.

Phase 1: This phase was conducted between 2017 and 2018 and applied a descriptive qualitative approach [13,14], exploring recommendations that pharmacy faculty trainees believed contributed to success of the Rx for Change program. A subset of randomly selected faculty trainees (n = 18 of the original 191 trainees, representing 16 pharmacy schools) participated in semi-structured telephone interviews, which were audio-recorded and transcribed. Fifteen of the participants were female; 15 were white, 2 were African American, and 1 was Asian. At the time of the training, the average age and years in their current position were 36.9 years (SD, 9.3; range, 27–56) and 4 years (SD, 4.8; range, 0–22), respectively. Most were assistant professors (n = 12), with 4 associate professors, 1 full professor, and 1 other. Two thirds were members of a pharmacy practice department, and one third identified as a member of the social and administrative sciences.

Qualitative analyses were conducted using an inductive approach with MAXQDA software [15]. Two investigators coded transcripts independently to identify relevant specific recommendations and then met to compare, discuss, and reach consensus. Once themes (i.e., recommendations) were identified, each recommendation was mapped onto one or more of the domains of Rogers’ Diffusion of Innovations Theory [11]. Further details on the purpose, methodology, and results of phase 1 were described previously [7]. This study was approved by the Purdue University Human Research Protection Program.

Phase 2: The findings from phase 1 informed the development of a web-based survey that was administered between 2019 and 2020 to the entire cohort of train-the-trainer participants [12]. The instrument estimated the impact of the workshops with respect to the following: (a) reach to pharmacy schools across the United States; (b) effectiveness on faculty confidence, their students’ confidence, and tobacco-cessation-related practices; (c) adoption of the Rx for Change materials for teaching tobacco cessation; (d) extent of implementation of Rx for Change in pharmacy schools and challenges faced; and (e) maintenance of the adoption of the Rx for Change materials for long-term use, using the RE-AIM framework [16]. The instrument also included items to assess additional perceptions of shared curricula such as cost-effectiveness and consideration for use in pharmacy schools. 

The phase 2 survey was estimated to take 15 min to complete. Because 15 or more years had elapsed since participating in a train-the-trainer program, extensive internet searches were conducted to locate individuals. Of 191 initial faculty participants, valid and current email addresses were identified for 137, and the survey was completed by 111 (81.0%), representing 75 (84.3%) of the 89 schools or colleges of pharmacy that participated in a train-the-trainer workshop [12]. Most (78.4%) currently held an academic position, 30.6% practiced in a clinical setting, 6.3% were retired, and 9.0% worked in a nonacademic, nonclinical setting (responses were not mutually exclusive). Descriptive analyses were conducted using SPSS software version 26 [17]. Further details on the methodology and results of phase 2 are available elsewhere [12]. This study was approved by the Purdue University Human Research Protection Program.

## 3. Results

### 3.1. Synthesis of Phase 1 and Phase 2 Studies

Faculty perceptions of the shared curricula concept, in general, are delineated in Table 1 and Table 2. Many participants (77.9%) who currently work in an academic setting agreed that shared curricula should be more broadly considered for use in pharmacy schools.

Across both phases, faculty participants described several aspects of the Rx for Change program that were perceived to be associated with program success. Seven core recommendations (Table 2) were identified in phase 1, five of which were further explored in phase 2. To reduce the overall length of the survey, two recommendations (“Meet accreditation standards” and “Demonstrate effectiveness”) were omitted from the phase 2 study, because both were deemed essential for any program that is to be disseminated within academia and therefore qualitative perceptions were regarded as sufficient. Each of the seven core recommendations identified in phase 1, and those that were further clarified in phase 2, are discussed below.

**Table 2:** Faculty perceptions of shared curricula in pharmacy education (% of n = 87 faculty members currently working in academia).

Characteristic	Agree	Neutral	Disagree
Shared curricula (in general) are a cost-effective approach to teaching.	79.0	17.4	3.5
Shared curricula should be more broadly considered for use in pharmacy schools.	77.9	18.6	3.5
Availability of a shared curriculum limits academic freedom.	16.3	12.8	71.0
Availability of a shared curriculum limits creativity.	24.4	15.1	60.5
Availability of a shared curriculum limits the feeling of “ownership.”	32.6	22.1	45.3

*Appeal to attendees:* Participants indicated a number of recommendations related to the Rx for Change program that were appealing and impacted their decision to attend the workshop. Specifically, the workshop provided an opportunity to bring new information about tobacco cessation back to their institutions, thus filling a gap in their curriculum. Other appealing attributes included the following: teaching materials were freely accessible online, travel costs to San Francisco were covered, and the program addressed a topic of personal interest. When asked in the survey what influenced their decision to attend the train-the-trainer workshop, the most highly rated reason was to improve their teaching of tobacco-cessation content.

*Relate content to clinical practice:* Participants indicated that Rx for Change had a high degree of relevance to clinical practice. Many mentioned that the “hands-on” activities were particularly helpful, including the counseling on the medications for cessation. They also indicated that motivational interviewing approaches, which involve a “*really complex set of skills*” (as described by a participant) help to improve patient counseling services in clinical practice.

Participants emphasized that all training programs should be relevant to practice and should facilitate attendees’ confidence and competence for teaching the material and for helping students apply the material. Participants acknowledged that implementing a new clinical service is challenging and therefore suggested that training programs should address how to successfully implement such services in practice settings.

*Deliver live training (in person):* Participants appreciated that Rx for Change was delivered as a face-to-face, live training program. They indicated that attending the program in person provided an opportunity to become “*really immersed*” in learning the material. They felt this format allowed for the use of a variety of methods of content delivery, including “*hands-on*” activities, decreased distractions, and opportunities to network and interact with other faculty members with similar teaching responsibilities and interests.

Several participants valued a blended workshop, with live, in-person training followed by web-based sessions delivered regularly. One participant explained, “*I personally like the live training, particularly for the first time that you’re going through it.*” Other participants, however, highlighted the advantages of web-based training programs including convenience (e.g., asynchronous delivery, elimination of travel, less time away from work) and the ability to reach a greater audience. Still other participants suggested that web-based training might not engage participants if trainees are not “*truly invested in learning.*” A participant commented, “*When you do any kind of web-based [training], it’s easy to not feel connected to the rest of the people in the group and lose motivation*.”

*Develop high-quality materials, delivered by experts:* Participants indicated that high-quality, evidence-based, turnkey (for implementation) materials contributed to the success of the Rx for Change program. They valued that experienced faculty with clinical expertise in teaching tobacco content delivered these workshops. Results from the survey confirmed that participants rated the quality of the Rx for Change curriculum highly, including the various “characteristics of the innovation” described by Rogers [11].

Participants suggested enhancements to the structure and content of future training programs, including how to deliver curriculum using “newer” methods. As one participant illustrated, “*…here’s a way of [implementing the curriculum using] team-based learning principles, here’s a way of doing it using online instruction, here’s the way of doing it in a flipped classroom. So, there is probably a wider range of methodologies that are being used to teach.*” Some suggested adding discussions on recently published studies, and others suggested adding discussions on controversial topics. Several participants suggested adding instructions on how to deliver the content under curricular time constraints: “*What [are] the best things to include if you had limited time? How to prioritize those things? Especially from a new faculty member’s perspective, it was all just a bit overwhelming…So if you don’t have the amount of time to [implement] everything or are overwhelmed with everything, what’s the best place to start?*”

*Provide support:* Participants appreciated the efforts devoted by the Rx for Change team to support faculty attendees. They described three types of perceived support. First, the availability of a website to access routinely updated teaching materials (https://rxforchange.ucsf.edu, accessed on 28 June 2023) [3]. In the phase 2 survey, 89% of 86 participants working in academia rated the Rx for Change website to be “very” or “extremely” useful for supporting teaching of tobacco cessation [12]. Second, participants valued that they were invited to the training with another colleague from the same institution, and this was perceived as a facilitator for implementation of the content. For example, one faculty member said: “*so definitely I and [my colleague] who did the training as well, she’s been a supporter.*” Third, participants appreciated the placebo cessation aids (lozenge, inhaler, and nasal spray) that were provided after the training, as it helped them to instruct students and patients on the proper use of these medications through hands-on demonstration.

*Meet accreditation standards:* In phase 1, participants shared that Rx for Change was successful because it met accreditation standards and addressed required competencies for pharmacy school curricula. As such, faculty could easily “pitch” the content to the curriculum committee at their institution.

*Demonstrate effectiveness:* Prior studies have demonstrated the widespread, sustained use [18] as well as the effectiveness of the Rx for Change program, including changes in clinical practice [19,20,21]. While the phase 1 interviewees did not mention that they were aware of published evidence demonstrating the effectiveness of the Rx for Change program, they did emphasize that training programs must show impact. Specifically, they mentioned four ways by which training programs should be evaluated: (1) determine whether the learning objectives were met, (2) determine whether the curriculum was successfully implemented at the trainees’ institutions, (3) estimate the impact on student outcomes using pre- and post-training surveys, and (4) conduct studies to estimate the distal impact on patients. While some of these are beyond of the scope of most train-the-trainer programs, each would enhance perceptions of programs that are being disseminated.

### 3.2. Faculty Recommendations for Future Shared Curriculum Initiatives

In phase 1, participants suggested health topics for which a shared curriculum would be useful, and these were further explored in phase 2. Participants rated the following as very or extremely useful as a shared curriculum topic: opioid dependence (82.9%); drugs of abuse, including but not limited to opioids (76.6%); medical marijuana (71.1%); motivational interviewing (67.5%); pain management (63.0%); alcohol abuse (63.9%); obesity (59.4%); and law/jurisprudence (44.1%).

## 4. Discussion

The objective of this analysis was to synthesize findings from a mixed-methods study to craft recommendations for future developers of shared curricula. The Rx for Change program provides a unique framework and model for this type of analysis because it has been in existence since 1999 and was disseminated nationally, through train-the-trainer faculty development programs, to 98% of the schools of pharmacy in 2005 [9].

Because it is an appropriate framework for exploring recommendations associated with the adoption of an innovation, Roger’s Diffusion of Innovations Theory guided the development and dissemination of the Rx for Change program, and results were mapped onto the domains of the theory [11]. Data indicate that the “relative advantages” of the Rx for Change faculty training workshop that were important from participants’ perspective were as follows: it appealed to attendees (recommendation 1), it was delivered live (recommendation 3), it was perceived to be an improvement over other materials for teaching tobacco cessation (recommendation 4), it provided ongoing support (recommendation 5), and it met accreditation standards (recommendation 6). Compatibility and complexity/simplicity were also important, in that participants perceived the curriculum to be high-quality and easy to implement (recommendation 4). Trialability of the program was evident because the program demonstrated effectiveness in other research studies described in the literature (recommendation 7) [19,20,21]. We concur with participants’ opinions regarding the need to demonstrate the effectiveness of newly developed shared curricula by evaluating the training workshop, adoption by institutions, impact on students’ learning and clinical practice, and impact on patients. Future curriculum developers are encouraged to refer to prior Rx for Change evaluation efforts [1,2,7,9,10,12,18,19,20,21,22,23,24]. Finally, observable results were described by faculty in terms of perceived impact on students’ competency, confidence, and readiness to apply the learned skills (recommendation 2).

This study adds important information regarding the development and dissemination of shared curricula to enhance adoptions and maintenance of those adoptions. Participants indicated that certain aspects were important in making a training workshop appealing to faculty attendees (recommendation 1). These include removing cost burdens, focusing on a topic that addresses an important gap in health professions education, and training faculty to acquire skills needed as educators. These findings are consistent with guidance provided by Yelon et al., who reported that educational skills training is important for the success of a faculty development program [25]. The pharmacy education literature suggests the importance of teaching curricula using engaging methods, such as “active learning,” rather than solely using traditional lecture-based approaches (recommendations 1 and 2) [26]. Active learning was intentionally incorporated into the development of the Rx for Change curriculum, and such approaches are recommended for similar future endeavors. Additionally, to promote the application of knowledge and skills learned, medical education researchers have established the importance of providing support to faculty participants (recommendation 5) [27,28]. When faculty trainees receive adequate support, they are more likely to use and apply what they have learned during their training [27].

Based on our results and the studies by Lupu et al. and Bookstaver et al., it is recommended that curricula be delivered in a way that boosts students’ confidence and competence to effectively apply what they learned in the classroom during actual patient encounters [29,30]. This also mirrors the Accreditation Council for Pharmacy Education’s (ACPE) requirements to enhance “knowledge application and practice competencies” among pharmacy students (recommendation 6) [31]. Our study participants also requested more guidance on the different pedagogical methods and guidance on prioritizing content when time is limited (recommendation 4). Although numerous train-the-trainer pharmacy-related educational programs (addressing a variety of health topics) were previously evaluated and reported significant success [5,32,33,34,35,36,37,38], none provided specific recommendations to sustain programs long-term. More recently, the recommendations herein are being applied toward the development of a shared opioid-use-disorder curriculum for pharmacy schools. Paralleling our approach used for tobacco cessation, the opioid effort involved surveys of pharmacy faculty [39], pharmacists in practice [40], and patients, as well as focus groups with healthcare providers.

Because participants were interviewed or surveyed at least 15 years after their workshop training, it is likely that they had forgotten some aspects of the program. Yet, this time lapse is also a strength because it was more likely that participants recounted only the most salient or memorable aspects of their experiences, whether these were positive or negative. However, because of the time that had elapsed, 26.3% of our participants were unable to be located for the phase 2 survey study [12], and this could have biased the results. Additionally, the recommendations for developing and disseminating shared curricula are made based on our experiences with Rx for Change within the pharmacy profession and therefore might not be generalizable to other health disciplines, clinical content areas, or initiatives that did not benefit from federal grant funding over the years. Another limitation is that data were collected prior to the COVID-19 pandemic; therefore, participants conveyed great appreciation for the in-person training programs. We believe that many parts of a high-quality training can be delivered online using the current technology, and some of the more hands-on training can be delivered to small groups while exercising caution and social distancing. However, the in-person design of the training programs was perceived by faculty as important for future endeavors. Given that the results pertain to one program and one discipline, results should be interpreted with caution. Despite limitations, the recommendations provide guidance to faculty who are considering the development of similar new initiatives.

## 5. Conclusions

This investigation indicates that faculty members view shared curricula as a cost-effective approach to teaching that should be more broadly considered for incorporation into pharmacy education. Results of this study provide evidence and guidance related to key recommendations that are likely to enhance the long-term success of shared curricula. To enhance participation and long-term engagement, future training program developers should consider the motivating factors that appeal to trainees. The sustainability of a program is likely to be enhanced if it includes practical application (hands-on) components in training workshops and high-quality, evidence-based materials that are developed and maintained by content experts. The validity of the guidance provided could be tested in the development of shared curricula for key topics identified, including opioid dependence, drugs of abuse, medical marijuana, and motivational interviewing. Future curriculum developers are encouraged to evaluate the effectiveness of their new program by estimating its impact on students and on patient care. Additionally, assessment is needed of (a) the extent of program adoption and (b) maintenance of adoption, over time, by institutions. The study’s findings contribute to the field of health professional education by identifying key factors that promote adoption and sustainability of shared curricula and by offering recommendations for future initiatives.

## Figures and Tables

**Table 1:** Qualitative findings (representative quotations from phase 1) and quantitative findings (survey responses from phase 2) related to key recommendations for successful shared curricula. The recommendations are mapped to Rogers’ Diffusion of Innovations Theory [11].

Phase 1 Recommendations		Phase 1: Representative Quotations	Phase 2: Quantitative Findings	Rogers’ Diffusion of Innovations Element
1	Appeal to attendees	*“I was interested in the [tobacco epidemic] topic and it was a great opportunity for me as a faculty member and for the school to start our students in the [Rx for Change] curriculum.”*	Reasons for attending a train-the-trainer program (% reporting very or extremely important): To improve teaching for tobacco cessation (86.2%) To improve tobacco content in our curriculum (78.7%) To improve skills for treating tobacco use and dependence (78.0%) To be part of this national initiative (57.3%) An opportunity to meet colleagues with similar interests (52.8%) Was encouraged by a mentor/colleague (47.5%) Was required or encouraged by university administration (41.9%) Opportunity to travel to San Francisco at no cost (17.4%)	Relative advantage
2	Relate content to clinical practice	*“When…confronting a patient about tobacco use, if you feel more confident and competent in the approach, you are more likely to use it.”*	96.4% perceived having students apply tobacco cessation-counseling skills during IPPE/APPE rotations to be very/extremely important 84.0% perceived the inclusion of tobacco-related questions on the NAPLEX examination to be very/extremely important 81.3% perceived the program to be very/extremely impactful on students’ competency for tobacco cessation counseling 78.1% perceived the program to be very/extremely impactful on students’ readiness to apply their knowledge in practice 73.6% perceived the program to be very/extremely impactful on students’ confidence for tobacco cessation counseling	Observable results
3	Deliver live training	*“We were all away from our primary place of work, really immersed in [the live training]. We were focused.”*	Perception that conducting live, on-site train-the-trainer workshops would be: Very/extremely effective (67.3%) Moderately effective (23.6%) A little/not at all effective (9.1%)	Relative advantage
4	Develop high-quality materials, delivered by experts	*“I realize all the hard work that went into developing the materials…they are top notch and of high-quality and it was always something that you could definitely implement knowing confidently that the materials were spot on.”*	Perceived the shared curriculum to have high (H), moderate (M), or low/none (L) ratings for: Relative advantage over other materials: 70.7% H, 22.8% M, 6.5% L Compatibility with existing curriculum structure: 71.2% H, 24.0% M, 4.8% L Simplicity of implementing Rx for Change: 74.1% H, 23.1% M, 2.8% L Other factors: Appropriateness of teaching methodologies: 80.7% H, 16.5% M, 2.8% L Comprehensiveness of content: 90.1% H, 9.9% M, 0% L	Relative advantage, compatibility, complexity/ simplicity
5	Provide support	*“The materials are updated frequently enough. The relevant information in terms of the pharmacotherapy options, videos, and case scenarios...every time I go to that website, at least annually but often much more frequently...it’s updated.”*	Perceived usefulness of the Rx for Change website: Extremely (49.4%) Very (40.5%) Moderately (8.9%) A little (1.3%)	Relative advantage
6	Meet accreditation standards	*“[The curriculum committee] has to value [the new curriculum]. It’s not just enough for a faculty member to say this is important. Everything has to be linked to a standard or a competency.”*	Not assessed in phase 2.	Relative advantage
7	Demonstrate effectiveness	*“You really want to know if it [the program: training and subsequent curriculum implementation] had an impact on individuals.”*	Not assessed in phase 2.	Trialability

Abbreviations. NAPLEX: North American Pharmacist Licensure Examination; IPPE: Introductory Pharmacy Practice Experiences; APPE: Advanced Pharmacy Practice Experiences.

## Data Availability

Not applicable.
